# Prevalence and risk factors for brucellosis in prolonged fever patients in post-conflict Northern Uganda

**DOI:** 10.4314/ahs.v18i1.4

**Published:** 2018-03

**Authors:** Harriet N Muloki, Joseph Erume, David O Owiny, Joseph M Kungu, Jesca Nakavuma, Duncan Ogeng, George W Nasinyama

**Affiliations:** 1 Faculty of Agriculture and Environment, Gulu University. P. O. Box 166, Gulu, Uganda; 2 College of Veterinary Medicine, Animal Resources and Biosecurity, Makerere University, P. O. Box 7062, Kampala, Uganda; Current Address: Kampala International University, P.O Box 20000, Kampala, Uganda; 3 National Livestock Resources Research Institute, P.O.Box 96, Tororo

**Keywords:** Brucellosis, human, fever, prevalence, Uganda, zoonosis

## Abstract

**Background:**

Brucellosis is a disease with significant public and economic implications but strategies for controlling this disease remain problematic.

**Objectives:**

This study sought to determine the sero-prevalence of brucellosis in prolonged fever patients and to identify modifiable risk factors for the infection in humans in post conflict Northern Uganda.

**Methods:**

The study employed a cross-sectional method among prolonged fever patients who had visited selected health facilities in the study districts in Northern Uganda. Sero-prevalence of brucellosis was calculated for i-ELISA IgG/IgM. A structured questionnaire was used to obtain data on possible risk factors for brucellosis. Associations between sero-prevalence and risk factors were measured using the Odds Ratio.

**Results:**

Brucellosis was confirmed in 18.7% of the 251 patients that tested positive for the disease, with the rapid Brucella Plate Agglutination Test, and ages 10–84 years (median age 47+0.86). Sex (p = 0.001; OR 3.79; 95% CI 1.75 – 8.24), rearing livestock (p < 0.005; OR 8.44; 95% CI 2.84–25.03) and consumption of unpasteurised milk (p = 0.023; OR 2.57; 95% CI 1.14–5.80) were factors associated with brucellosis.

**Conclusion:**

Control of brucellosis in animals, training and sensitisation of the community on brucellosis is needed to stimulate action on human brucellosis control.

## Introduction

Brucellosis is a widespread zoonosis of both public health and economic concern^1^–^3^. The global burden of human brucellosis remains enormous with more than 500,000 new infections occurring annually worldwide^4^. Brucellosis is caused by organisms of genus *Brucella* with six pathogenic species of which four are known to cause human disease^5^. *B. melitensis* cause the most severe human disease, followed by *B. suis, B. abortus* and *B. canis*^6^. In humans brucellosis is transmitted by direct or indirect contact with infected animals and their products^7^. Inhalation of infectious aerosols and ingestion of raw milk or unpasteurized dairy products or meat from an infected animal is the most frequent route of infection^8^,^9^. Major symptoms for brucellosis in humans include; undulant fever, weight loss, back pain, fatigue and night sweats^1^.

Diagnosis of brucellosis is impossible without laboratory confirmation involving a combination of methods^10^. Blood culture for *Brucellae* isolation remains the gold standard^11^ but may not provide a positive result for all patients^12^. Nucleic acid amplification can provide rapid detection and confirmation of *Brucella*^13^ but standardization of extraction methods, infrastructure, equipment and expertise are still lacking^6^. Bacteriological and genomic limitations make serology the most practical and useful tool for brucellosis diagnosis^11^. The serological tests like Rose Bengal Plate Agglutination Test (RBPT), standard tube agglutination test (STAT), Enzyme Linked Immuno-Sorbent Assay (ELISA) and fluorescence polarization assay (FPA) among others have been applied in human brucellosis diagnosis^11^,^13^,^14^. However, STAT remains the most popular serological test used^14^ and titres of 1:160 and above are considered diagnostic^13^. Nevertheless, STAT has limitations making ELISA to be most acceptable for diagnosing human brucellosis^11^,^15^,^16^. Mantur and others^11^ found ELISA to be more sensitive than STAT in detecting brucellosis in both acute and chronic cases while sensitivity and specificity of ELISA was reported to be 71.3% and 100% respectively. Araj and others^15^ reported sensitivities of 100% and 91% for IgM and IgG ELISAs, respectively, and a specificity of 100% for both assays. In addition, Asaad and other workers^16^ reported sensitivities of ELISA IgM and IgG to be 85.2% and 96.3%,respectively and specificity of 100% for each..

Human brucellosis occurs widely in Uganda^8^,^17^,^18^. A recent study carried out in Gulu district in Northern Uganda reported individual animal and herd level sero-prevalence of bovine brucellosis to be 6% and 19%, respectively. Restocking and communal grazing were the major risk factors associated with the disease^19^. Northern Uganda is under rehabilitation with promotion of livestock rearing and re-stocking. To date, no study has described human brucellosis in post conflict Northern Uganda, a region that witnessed a total breakdown in socio-economic infrastructure, governance and services. The aim of this study was to estimate the sero-prevalence of human brucellosis in prolonged fever patients and identify modifiable risk factors for the infection in post-conflict Northern Uganda. This study considered ELISA IgG/IgM which is reported as the most specific serology test against SAT.

## Methods

### Ethics and consent to participate statement

Ethical approval was obtained from Gulu University Research Council (Ref No: GU/IRC/02/07/13) and the Uganda National Council of Science and Technology (UNCST) Reference No: HS 1442. Written consent/assent was sought from all individuals before enrolment.

The study was carried out in post-conflict areas of Northern Uganda, in the districts of Apac, Gulu, Lira and Pader covering up to nine percent of the country's 236,040 Km^2^ area size.

### Study design and enrolment of study patients

A cross-sectional study was conducted from March 2014 to February 2015. Study patients were selected from 17 out of the 244 public and private health facilities in the study area^20^–^23^. The health facilities were purposively selected basing on capacity to test for brucellosis and willingness to participate in the study. The study targeted patients with fever, headache, joint pain, malaise, backache, fatigue and loss of appetite visiting study health facilities. Medical personnel in the out patient department (OPD) identified suspects and tested them with the rapid Brucella Plate Agglutination Test (BPAT). The sample size for study subjects was calculated using the formula for cross sectional survey as described before^24^ at 5% desired precision and 95% confidence interval with prevalence of human brucellosis in Uganda estimated at 18%^8^.

### Laboratory investigation

To determine the sero-prevalence of brucellosis in prolonged fever patients, STAT 1: 160 was used as the standard serological test against SAT 1: 320, iELISA IgG, iELISA IgM and iELISA IgG/IgM. Only BPAT positive samples from consented subjects were further investigated. All subjects were briefed on the objectives of the study, and signed a consent form. Cephalic vein blood was collected into sterile silicon-coated tubes without anticoagulant for serum harvesting after 24 hours at room temperature. The serum was transported on ice to Makerere University, College of Veterinary Medicine, Animal Resources and Biosecurity (CoVAB) and kept at -20°C. Samples were analysed using STAT (Remel kit, Europe) and i-ELISA (Vircell S.L, Santa Fe, and Granada, Spain) according to the manufacturers' guidelines. To improve sensitivity, results for i-ELISA, were interpreted in parallel, with any IgG or IgM positive test reported as positive for i-ELISA as described previously^17^. A structured questionnaire was used to obtain data on possible risk factors for human brucellosis. Information collected from study subjects included age, gender, district of origin, education level, rearing livestock, knowledge on brucellosis transmission and practice of consumption of unpasteurized milk/milk products.

### Statistical analysis

Sero-prevalence of brucellosis in humans was calculated for both STAT and i-ELISA (IgG and IgM). Associations between sero-prevalence of human brucellosis and modifiable risk factors were assessed with a Chi-square test and Odds Ratio at 95% confidence interval using SPSS, (SPSS Inc. Chicago, USA Version 16.0). Variables with p ≤ 0.1 at univariable analysis were included in a multivariable logistic regression model. The backward selection procedure was used to identify factors for the final models. Goodness of fit for the final model was assessed using Hosmer and Lemeshow goodness of fit test.

## Results

**Figure 1:**
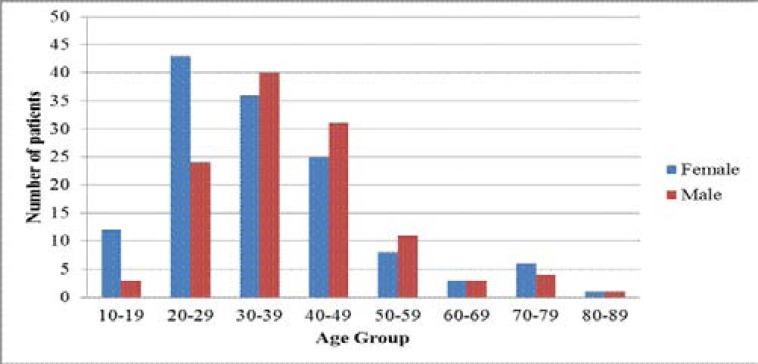
Distribution of study patients by gender and age group

A total of 251 prolonged fever patients aged 10–84 years (median age 47.0± 0.86) were enrolled in the study (Fig 1).

Up to 207 study subjects were from government and 44 from private health facilities. All serological tests showed significant (p<0.05) differences in brucellosis sero-positivity between health facilities, sex and keeping livestock. In addition brucellosis sero-positivity differed significantly (p<0.05) with consumption of milk and milk products basing on STAT 1:160, 1:320 and i-ELISA IgM, but did not differ basing on i-ELISA IgG. There was no difference in sero-positivity with education level (p>0.05) basing on all the four serological tests (Table 1).

**Table 1:** Sero-prevalence of brucella among prolonged fever patients

			Test Results (%,p-value), n=251			
Variable	Category	Frequency	STAT 1:160	STAT1:320	i-ELISA IgG	i-ELISA IgM
Health facility	Government	207	32.7(0.000)*	17.5(0.026)*	12.7(0.005)*	10.8(0.039)*
	Private	44	2.0	1.2	0.0	0.4
Gender	Male	117	19.9(0.012)*	11.2 (0.048)*	10.8(0.000)*	8.4(0.001)*
	Female	134	14.7	7.5	1.9	2.8
Age category of patients	Children	8	0.8(0.887)	0.0(0.0507)	0.0(0.722)	0.0(0.361)
	Youth	188	26.3	14.3	9.6	7.6
	Adult	41	6.0	3.6	2.4	2.4
	Elderly	14	1.6	0.8	0.8	1.2
Educational	≤Primary	152	22.7(0.242)	11.2(0.878)	7.6(0.883)	8.0(0.212)
level	> Primary	99	12.0	7.5	5.1	3.2
Keep livestock	Yes	145	29.9(0.000)*	16.3(0.000)*	11.1(0.000)*	10.4(0.000)*
	No	106	4.8	2.4	1.6	0.8
Knowledge Transmission	No	231	31.5(0.601)	16.7(0.453)	11.6(0.753)	9.2(0.040)*
	Yes	20	3.2	2.0	1.1	2.0
Consume pasteurised Milk/milk products	No	151	27.1(0.000)*	17.1(0.000)*	9.6(0.066)	9.2(0.012)*
	Yes	100	7.6	1.6	3.1	2.0

* 5% level of significance

Basing on the parallel interpretation of either positive i-ELISA IgG or IgM or both tests, brucellosis sero-prevalence differed significantly (p<0.05) with gender, rearing livestock and consumption of unpasteurised milk/milk products (Table 2).

**Table 2:** Relationship between the hypothesised risk factors for human brucellosis and sero-positivity based on i-ELISA IgG/IgM.

Variable	Category	% test positive (n=251)	χ^2^	df	p-value
Gender	Male	14.3	20.89	1	<0.005*
	Female	4.4			
Age group	< 30years	5.2	3.15	1	0.076
	> 30 years	13.5			
Education level	≤Primary	12.0	0.26	1	0.611
	> Primary	6.7			
Keep livestock	Yes	17.1	26.95	1	<0.005*
	No	1.6			
Knowledge on	Not Know	14.3	1.82	1	0.178
Transmission	Knowledgeable	3.4			
Consume milk	Unpasteurised	14.7	8.32	1	0.004*
and milk products	Pasteurised	4.0			

* Significant at 5% level

Gender (OR 3.79; 95% CI: 1.75–8.24), livestock rearing (OR 8.44; 95% CI: 2.84–25.03) and consumption of unpasteurised milk (OR 2.57; 95% CI: 1.14–5.80) were significantly associated with brucellosis. The model explained 30.9% (NagelkerkeoR2) of the variance in brucellosis sero-prevalence and correctly classified 83.3% of the cases. The Odds of being brucella positive in males with prolonged fever were four times the Odds in females. Livestock rearing was highly associated with brucella sero-positivity and consumption of unpasteurised milk and milk products increased the likelihood of testing brucella sero-positive (Table 3).

**Table 3:** Multivariable logistic regression of factors associated with human brucellosis in post-conflict northern Uganda

Variable	Category	% Sero- positivity (n=251)	Odds Ratios(95%CI)	p-values
Gender	Male	14.3	3.79(1.75–8.24)	0.001*
	Female	4.4		
Age group	< 30 years	5.2	1.73 (0.80–3.75)	0.164
	> 30 years	13.5		
Keep livestock	Yes	17.1	8.44(2.84–25.03)	0.005*
	No	1.6		
Consumption of	Unpasteurised	14.7	2.57(1.14–5.80)	0.023*
Milk/milk products	Pasteurised	4.0		

* 5% level of significance

## Discussion

This is the first study to document the sero-prevalence of human brucellosis among patients with prolonged fever in Northern Uganda. The study showed sero-prevalence of human brucellosis in Northern Uganda to be 18.7% with i- ELISA and 34.7% with STAT (1:160). However, sero-positivity with STAT titres (1>1:320) was 18.7%, an indication of active infection. This is not surprising since study subjects had come to seek medical attention with symptoms relating to brucellosis. This finding is comparable to a recent study^17^ which reported STAT sero-prevalence results not to be different (p<0.05) from that for c-ELISA. Findings here support the use of ELISA tests for confirming brucellosis in low-income countries where brucellosis is endemic. This study showed men to be four times likely to test positive for brucellosis than women. In the current study, milking and grazing animals is a man's role thus men are more in contact with animals than women. This finding is supported by reports of epidemiological studies in other parts of the world where human brucellosis is considered an occupational disease^25^. In addition, reports show no sex-wise discrimination of brucellosis infection between male and female as both have equal susceptibility if provided with exposure to potential risk factors^26^. Nevertheless, the current study contradicts studies elsewhere which reported high prevalence of brucellosis among females than males^25^,^27^.

This study clearly shows livestock rearing (p<0.005) to be strongly associated with human brucellosis. Generally, livestock harbour brucellosis in a sub-clinical state and farm families are at high risk of exposure to the disease through contact with fluids of affected livestock or consumption of their unpasteurized products. This study is in agreement with Corbel^6^ who pointed out that when animals are kept in close proximity to human habitat, the practice can result in brucella infection as people are in constant contact with infective materials. This study has also shown brucellosis sero-positivity to be highly associated with consumption of unpasteurised milk/milk products. This finding parallels another study^28^ which reported that significantly (p<0.02) more sero-positive humans with a history of raw milk. This calls for increased awareness among the communities in post-conflict Northern Uganda about the dangers associated with drinking raw milk, among others. Since this study dealt with only suspected brucellosis patients and did not cover all health facilities, results need to be interpreted cautiously. However, the study has provided important information to support brucellosis control programs.

## Conclusion and recommendations

Findings in this study show clearly that the sero-prevalence of human brucellosis among patients with prolonged fever is high (18.7%). Rearing livestock and consumption of unpasteurised milk/milk products were factors associated with brucellosis. Strategies to control animal brucellosis and raising awareness about the consumption of unpasteurised milk and milk products are necessary to reduce the incidence of human infection in Northern Uganda. Innovative methods to sensitize the community on brucellosis and cost effective methods to control animal brucellosis are recommended.
